# Polydopamine coated shape memory polymer: enabling light triggered shape recovery, light controlled shape reprogramming and surface functionalization†

**DOI:** 10.1039/c6sc00584e

**Published:** 2016-04-12

**Authors:** Zhen Li, Xiaoyong Zhang, Shiqi Wang, Yang Yang, Benye Qin, Ke Wang, Tao Xie, Yen Wei, Yan Ji

**Affiliations:** a The Key Laboratory of Bioorganic Phosphorus Chemistry and Chemical Biology, Department of Chemistry, Tsinghua University Beijing 100084 China weiyen@mail.tsinghua.edu.cn jiyan@mail.tsinghua.edu.cn; b State Key Laboratory of Chemical Engineering, Department of Chemical and Biological Engineering, Zhejiang University Hangzhou 310027 China

## Abstract

Photo-active shape memory polymers (SMPs) are considered as a promising candidate for converting light into mechanical energy. However, most known SMPs are only thermo-responsive. To achieve photo-activity, photo-responsive choromophores or fillers usually have to be incorporated from the very beginning of the material synthesis. Here, we introduce a novel post-synthesis approach to endow normal SMPs with photo-active properties using mussel-inspired surface chemistry. Without changing the original properties, the resultant polydopamine (PDA) coated SMPs show an efficient photo-active performance. The coating can be easily patterned and erased, which allows flexible light-triggered 3-D shape deformation of a planar SMP sheet. Moreover, owing to the high chemical activity, the PDA coating also provides a platform to optimize the surface properties of the photo-responsive SMPs through secondary surface modification.

## Abstract 2

Simple dip-coating transforms thermally responsive shape memory polymers into photo-responsive materials and allows for shape engineering and surface functionalization.

## Graphical abstract

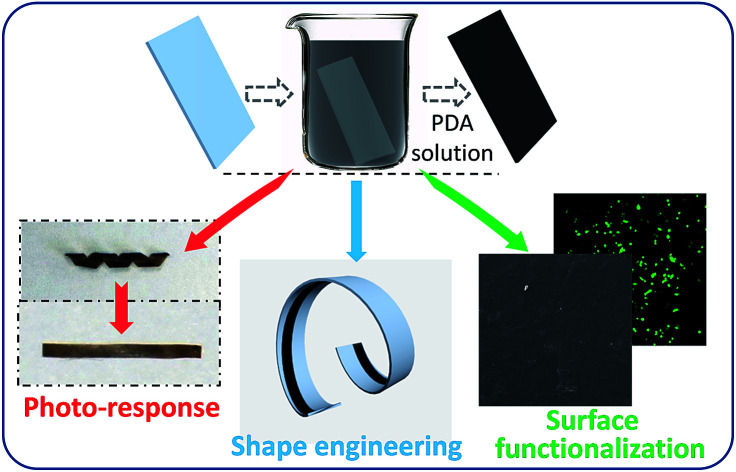

## Introduction

Smart materials capable of converting an input stimulus into output mechanical energy are of considerable and pervasive interest for many high-valued applications.^1–3^ As a suitable candidate, shape memory polymers (SMPs) attract particular attention since they can recover memorized original shapes in the presence of external triggers with substantial mechanical response.^4^ Owing to such properties, SMPs have been applied to or showed great promise in various fields from biomedical to textile materials.^5^ Most SMPs are inherently thermo-responsive. They change shape when heated to above a certain transition temperature (*T*_trans_). However, as an energy source, heat is hard to manipulate under actual working conditions. Compared to heat, light is a much more ideal stimulus since it can be applied remotely, focused accurately and switched rapidly.^6^ That's why there is a constantly growing interest in creating photo-active SMP actuators. Currently, most approaches to achieve photo-responsive SMPs can be generalized as either the introduction of photo-isomerisable chemical groups or the incorporation of light-absorbing additives based on a photo-thermal effect.^7–9^ No matter which approach it is, more often than not, the photosensitivity has to be designed into materials from the very beginning of the material synthesis.

Here, we present a post-synthesis approach to obtain photo-active SMPs. One simple, cost-effective and totally green dip coating procedure can turn normal widely-existing thermo-responsive SMPs into photo-responsive materials. The entire dip-coating process requires no organic solvents or toxic reactants and is accomplished in water solution. Moreover, the photo-responsivity can be erased by simply washing off the coating with base solution. This property, which could not be achieved in previous light-responsive SMPs, provides another level of versatility in the application of SMPs. This approach is based on the surface chemistry of a mussel-inspired polydopamine (PDA) coating, which was firstly disclosed by Messersmith *et al.*^10^ Dopamine was identified to play an important role in the marine mussel adhesive protein. Under weakly basic conditions, dopamine can undergo non-covalent self-assembly and oxidative self-polymerization. Further deprotonation and an intermolecular Michael addition reaction lead to the formation of a cross-linked polydopamine homopolymer.^11^ Owing to the catechol group, the polydopamine coating shows great adhesive ability for almost all materials, which is worth mentioning as an extraordinary feature. Till now, the polydopamine coating has been extensively utilized to modify multifarious materials with various functions.^12–18^ Recently, it was found that dopamine–melanin colloidal nanospheres could act as a novel photothermal therapeutic agent for cancer therapy, because these nanospheres are able to convert light into heat with an efficiency 100 times larger than that of carbon nanotubes.^19^ Surprisingly, few works have explored such a photothermal effect besides this report. We assume and verify here that, other than PDA nanospheres, PDA nanocoatings also serve as an excellent photothermal layer. Such layers can generate enough heat and trigger the phase transitions of normal SMPs even though the thickness of such layers is normally less than 50 nm.^10,13^ This allows for the expansion of the applications of the present thermo-responsive SMPs by just simple dip-coating.

In addition to light triggered shape recovery, this approach also allows us to explore light-induced shape engineering by utilizing the PDA pattern. Recently, a new shaping strategy based on chemical patterns was introduced to fabricate complex 3-D shapes from a flat sheet.^20–23^ Due to the difference in crosslinking density or chemical components, the pattern can guide the material to undergo a shape transformation into a pre-programmed 3D structure, which is difficult to obtain using traditional polymer shaping techniques such as molding. SMPs have been used as a substrate for photo patterning.^24–27^

However, most of the previous reports focus on the surface topology (such as wrinkles or arrays) rather than the macroscopic shape.^27,28^ Besides, these approaches can only be applied to certain materials with a special chemical design. Moreover, the patterns were usually not reprogrammable. Here, by regulating the PDA pattern, near infrared (NIR) irradiation can trigger different 3-D shape deformations from a pre-stretched planar sheet. And since PDA can be easily erased with alkali solution, this pattern is totally reprogrammable.^29^ The above photo-pattern also presents a light-manipulated shape programming ability, which provides more flexibility in the potential applications of SMPs. It is possible for the existing photo-responsive SMPs to undergo photo-programming through spatial control or patterned light exposure spots. The method used here is different as global irradiation can be used.

Furthermore, this approach provides a good opportunity for expanding the applications of existing SMPs through convenient secondary reactions. Apart from the ease of preparation and strong interfacial binding forces, the PDA layer stands out for its high chemical reactivity.^10,12^ PDA coated surfaces can be further modified through reactions such as Michael addition^30^ and Schiffbase formation^31^ by dip-coating with a thiol- or amine-containing solution. Therefore, the surface of SMPs can be altered to overcome some of the inherent limits of SMPs so as to accommodate different applications, as the application of an engineering material is strongly related to its surface properties.^32–34^ We demonstrate this by immersing the PDA coated SMPs into polylysine (PL) solution to improve cell compatibility and bovine serum albumin (BSA) solution to enhance blood compatibility. Clearly, this offers the possibility, at least to some extent, to utilize existing non-bio SMPs for biodevices without the need to synthesise new ones. Similarly, other kinds of surface modification can be done with this PDA coating.^35,36^ Other opaque layers, such as carbon coatings and gold nanocoatings, are also reported to show a photo-thermal effect. Compared to them, the approach presented here is easy to follow using green surface chemistry and the coating is capable of secondary modifications.^37^

## Results and discussion

### PDA nanocoating on the matrix

**Fig. 1:**
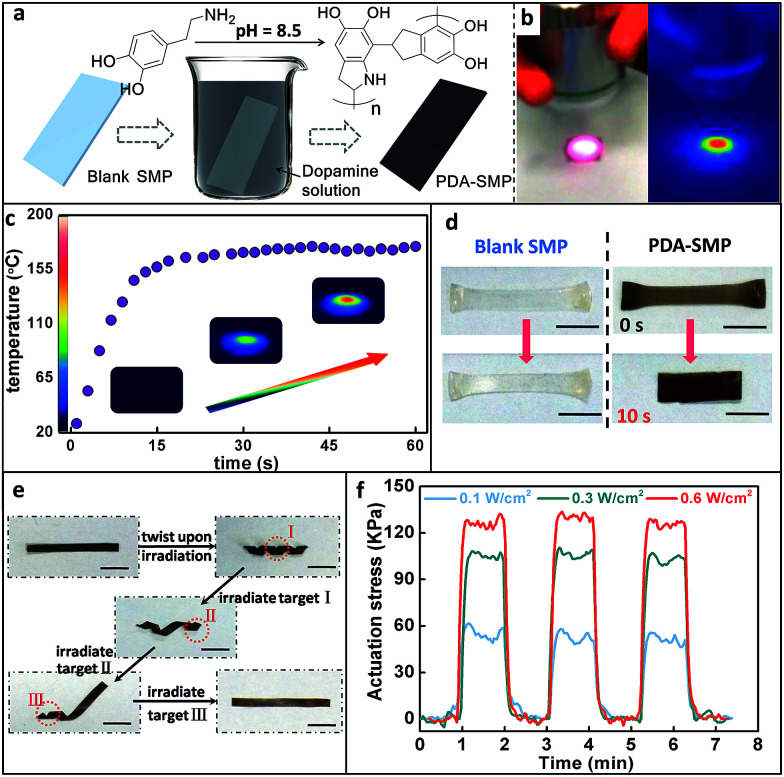
(a) Schematic diagram of the preparation process for a PDA-SMP; (b) optical and infrared thermal images of a PDA-SMP film irradiated with light. (c) Local temperature increase and IR thermal image of a PDA-SMP film under IR irradiation. (d) One-way photo-induced shape memory demonstration for a blank SMP film and a PDA-SMP film. (e) Photo control of the localized shape recovery of a PDA-SMP film. (f) The actuation stress of an aligned PDA-SMP sample irradiated at various light intensities. Film thickness: 0.15 mm. Scale bars: 0.5 cm.

A special shape memory polymer was used here as an example to demonstrate the strategy. This shape memory polymer is a diphenyl based liquid crystalline network.^38^ It is thermo-responsive and can perform the normal one-way shape memory and reversible actuation due to an LC-isotropic phase transition as previously reported by our group.^38^ PDA was formed on the surface of blank SMPs *via* the classical self polymerization of dopamine in a weak alkaline aqueous solution, as schematically represented in Fig. 1a.^10^ Infrared spectroscopy (IR) and X-ray photoelectron spectroscopy (XPS) were performed to confirm the existence of the PDA nanocoating on the resultant PDA coated SMPs (PDA-SMP). The optical absorbance spectrum shows that the PDA coating exhibits strong absorption in the whole visible optical range (ESI Fig. S1†). The thickness of the PDA coating was measured using atomic force microscopy (AFM) and scanning electron microscopy (SEM) as about 30 nm (ESI Fig. S2–3†). The PDA nanocoating doesn't change the original mechanical properties of the SMP. Differential scanning calorimetry (DSC), thermogravimetric analysis (TGA) and tensile storage modulus tests show that the thermal and mechanical properties of the PDA-SMP are almost the same as those of the blank one (ESI Fig. S4–6†).

### Photo-thermal effect and photo induced shape recovery

The light source used in this paper was an 808 nm near infrared (NIR) laser. In order to quantitatively understand the photo-thermal behavior, the temperature of the PDA-SMP sample was recorded using an infrared thermal imager. As shown in Fig. 1b, the red color indicates a higher temperature while blue represents a lower temperature. Under 1.4 W cm^−2^ irradiation, the temperature reached 160 °C (Fig. 1c) in 20 seconds, which is sufficient for an LC-isotropic phase transition (110 °C). The shape recovery can be easily achieved through light irradiation. This was first attested by one-way shape memory experiments.^39^ As shown in Fig. 1d, a blank and a PDA coated sample were both elongated at 120 °C (above Ti) and cooled down with this temporary shape. Then these two samples were irradiated from the top at an intensity of 1.4 W cm^−2^ for 10 s. The PDA coated sample recovered to its original size while the blank sample was not responsive to light at all, which clearly proved the strong photothermal effect of the PDA coating. As another example, spatially controlled shape recovery was able to be achieved. Firstly, the PDA-SMP was programmed into a helix (Fig. 1e). Irradiating the selected areas (target I, target II and target III) in sequence led to the unfolding of the sample step by step. In contrast, such remote and spatial control of the shape memory effect cannot be applied to the blank samples. Thirdly, reversible photo actuation can be achieved with an aligned SMP sample (a two-dimensional X-ray image is shown in ESI Fig. S7a†). As we previously reported,^38^ the liquid crystal units of this material can be oriented and fixed, resulting in a mono-domain sample. The mono-domain sample can display reversible thermal actuation as the sample goes through the LC-isotropic phase transition.^40,41^ When a mono-domain sample is prepared as previously reported and then coated with PDA, this actuation can be triggered by light. The PDA-SMP film contracted upon irradiation and returned to its original length when the light was switched off (ESI Fig. S7b and c†). This reversible photo actuation was further examined with a dynamic mechanical analyzer (DMA) in an iso-strain mode in which the strain was stabilized at 0% and the stress was recorded. As displayed in Fig. 1f, when the light was applied to the PDA-SMP sample, the stress increased rapidly and reached a constant value. When the light was switched off, the stress dropped back to zero within seconds. Its dependency on the light intensity was also evaluated. The light was switched on and off 3 times at a different intensity (0.1 W cm^−2^, 0.3 W cm^−2^ and 0.6 W cm^−2^) to test the reproducibility of the response at the same time. At each intensity, the actuation stress was approximately the same in all 3 cycles, and when the light was off, the stress went back to 0 kPa. Moreover, as the light intensity increased, the actuation stress increased as well.

### Light manipulated shape programming

**Fig. 2:**
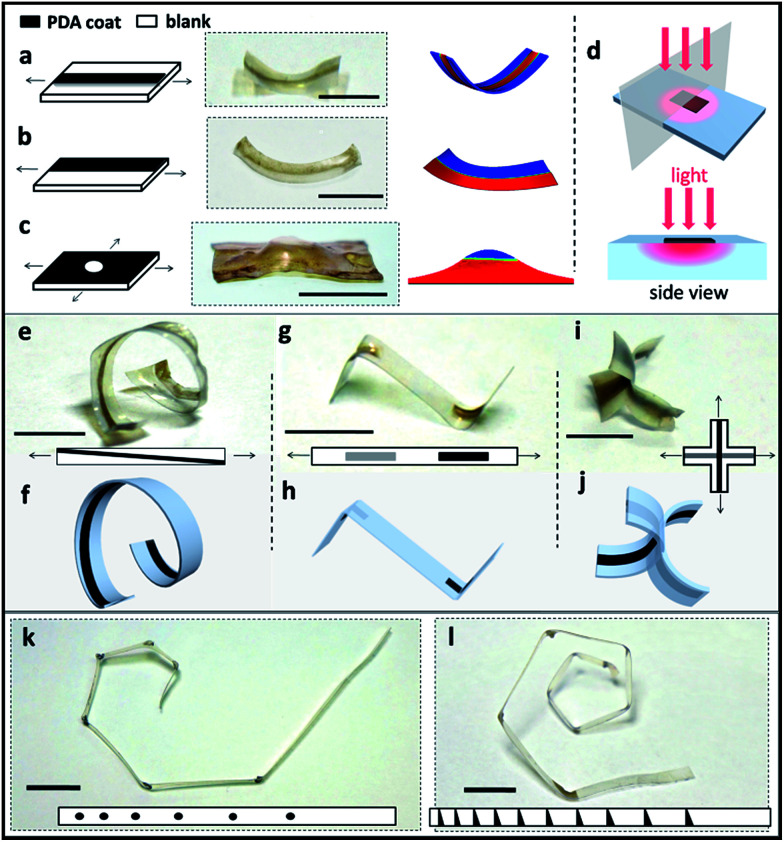
(a–c) Schematic representation (left), sample transformation photograph (middle) and FE model (right) of the PDA-SMPs with different patterns: (a) a PDA line coated in the middle of a uniaxially stretched sample; (b) a partially coated axially stretched sample; (c) an orthogonally stretched sample covered with a PDA coating but with an uncoated center. (d) Schematic representation of the photo-thermal mechanism for PDA-SMPs. (e and f) Photograph and schematic illustration of helix deformation. (g and h) Photograph and schematic illustration of double bending deformation in two directions. (i and j) Photograph and schematic illustration of a cross shape. (k) Spiral deformation in the plane guided by a pattern of PDA dots. (l) 3D spiral shape with increasing radius guided by a pattern of PDA triangles. To obtain these shapes, the PDA pattern areas of all the samples were irradiated with a 1.4 W cm^−2^ 808 nm laser for 15 s. Film thickness for 2c and 2i: 0.20 mm, others: 0.15 mm. Scale bars: 1 cm.

In order to program the material into complex 3-D structures, PDA patterns were made onto a flat pre-stretched SMP sheet. Using a Teflon mask, PDA was only deposited onto the exposed regions of the SMPs, leading to certain patterns (ESI Fig. S8†). Upon irradiation, the PDA coating converts light to heat. For the pre-stretched sample, the PDA-patterned regions are heated and tend to contract while the blank parts remain in the elongated state, which drives and guides the corresponding shape deformations. For example, as can be seen in Fig. 2a, a single PDA line patterned in the middle of the pre-stretched SMP surface guides the sample bending upon light irradiation. It is found that the bending direction is always towards the PDA coated side. We assume the reason for this phenomenon is that the temperature of the top and bottom of the sample is different for the first several seconds due to the heat transfer process, which causes contractions of different degrees along the film thickness and leads to bending in a certain direction. The temperature difference for the top and bottom of the PDA coated samples was proven by recording their temperature increment process, which is presented in Fig. S9.† It can be seen that the difference in temperature exists between the top and bottom of the film during the first 15 s of irradiation. A finite element (FE) model (Fig. 2a right) was used to study this situation and the result is consistent with our hypothesis. Further investigation and more examples are presented and discussed in ESI Fig. S10 and 11.† Similarly, the PDA pattern across half of the surface led to a bend towards both normal and side directions (Fig. 2b). In another case, as shown in Fig. 2c, a biaxially stretched SMP film totally coated with PDA except for the center point leads to a bulge. These shape deformations were also confirmed by the FE model. The mechanism of the PDA-pattern guided deformation is illustrated in Fig. 2d. Various 3-D shapes can be achieved by applying these simple patterns. An oblique line of PDA can give rise to a helix shape, which is shown in Fig. 2e and schematically presented in Fig. 2f. PDA can be patterned on both the top and bottom side of the SMP sample for more structures. As shown in Fig. 2g and h, a rectangular PDA pattern on the top and the same pattern on the bottom lead to the two parts of the sample bending in opposite directions. Similarly, patterning a biaxially stretched cross with a middle parallel PDA line on the top and a vertical PDA line on the bottom resulted in the shapes shown in Fig. 2i and j. More complex 3-D structures can be realized with more complex patterns. For example, in Fig. 2k, PDA dots can guide the sample into a 120° bend and a series of PDA circular dots can induce a planar spiral. In addition, a series of PDA triangles encouraged the formation of a 3D spiral structure as the sample bent in both normal and side directions (Fig. 2l). The radius of the spiral can be adjusted by changing the distance between the circular or triangular PDA dots.

### Shape reprogramming

**Fig. 3:**
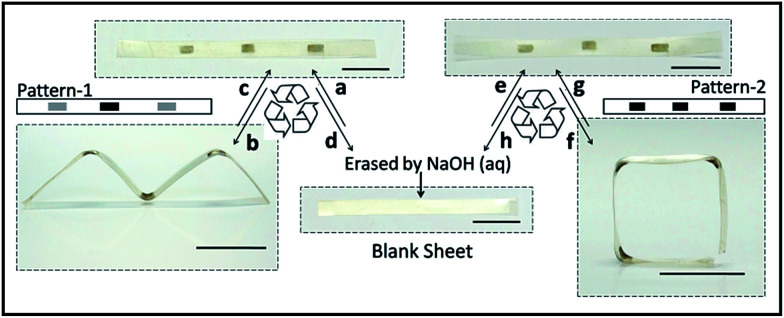
Shape-reprogramming. (a) Blank SMP was stretched to *ε* = 100% at 120 °C and then patterned with pattern-1 in dopamine solution (the black rectangle represents the top side and the gray rectangle represents the bottom side). (b) Irradiation of the patterned sample for 15 s. (c) Heating for 15 s at 120 °C while stretching to recover the flat shape. (d) Erasing the PDA pattern with 0.1 M NaOH aqueous solution. (e) Patterned with pattern-2 in dopamine solution. (f) Irradiation of the patterned sample for 15 s. (g) Heating for 20 s at 120 °C while stretching to recover the flat shape. (h) Erasing the PDA pattern with 0.1 M NaOH aqueous solution and back to the blank sheet. Light intensity: 1.4 W cm^−2^. Scale bars: 0.5 cm.

The PDA patterns are reprogrammable since PDA can be easily washed off with alkali liquor due to the depolymerization of PDA.^42^ As presented in Fig. 3, after patterning with pattern-1, the pre-stretched film deformed to an “M” shape (Fig. 3b) under NIR irradiation. Then the resultant sample was recovered to the initial shape by re-stretching at 120 °C, after which pattern-1 was erased with 0.1 M NaOH aqueous solution and re-patterned with pattern-2. Under irradiation, the sample performed a new shape transformation as shown in Fig. 3f. This experiment shows the reprogrammability of the PDA pattern, which enhances the versatility of this 3-D deformation technique.

### Surface functionalization

**Fig. 4:**
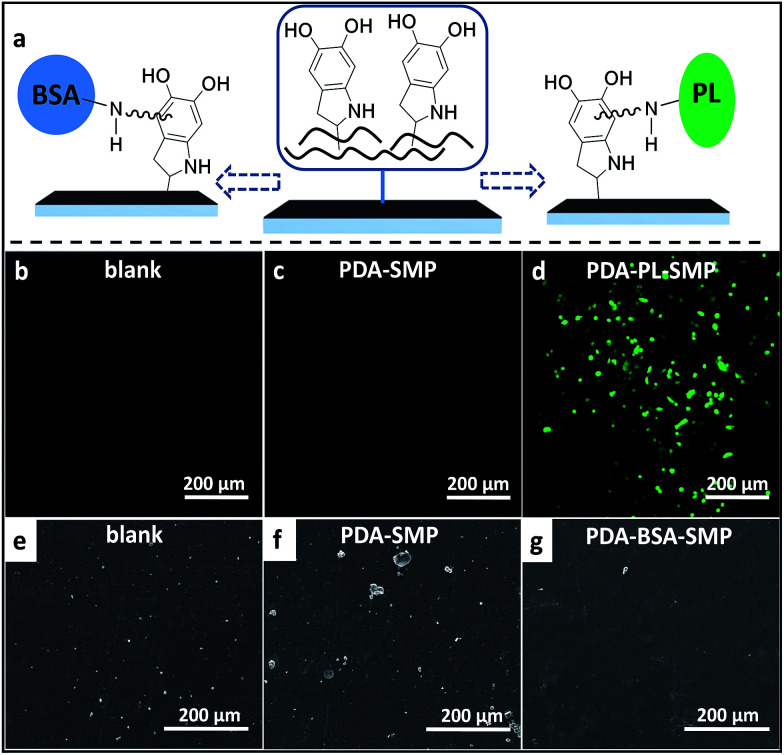
(a) Schematic diagram of the secondary modification of the PDA coating *via* Michael addition. (b–d) Confocal microscopy images of the A549 cells cultured on the surface of blank SMP, PDA-SMP and PDA-PL-SMP samples; (e–g) SEM images of rat blood platelet adhesion on the surface of blank SMP, PDA-SMP and PDA-BSA-SMP samples.

As a preliminary exploration on the feasibility of modifying the surface properties of PDA-SMPs through secondary surface modification so as to extend the application of SMPs, we carried out two qualitative tests, targeting the future bio-application of SMPs as cell substrate and implant devices. The schematic diagram of the modification mechanism is shown in Fig. 4a. For example, bovine serum albumin (BSA) and polylysine (PL) can be immobilized on the PDA coating through the addition of amino-groups to the α-carbons on the benzene rings. Firstly, we improved the cell viability of the PDA-SMPs by incubating the samples with polylysine (PL) aqueous solution at pH 9.5. The successful immobilization of PL on a PDA-SMP was confirmed from the XPS data (ESI Fig. S12†). A549 cells were transfected with green fluorescent dyes and cultured onto the surface of a PDA-SMP, blank SMP and PL modified PDA-SMP (PDA-PL-SMP). Normally, if the surface of the substrate is of good cell viability, the cells can adhere onto the surface and proliferate, otherwise the A549 cells may fail to multiply or adhere at all. As shown in Fig. 4b and c, no cell attachment was observed when the A549 cells were incubated with the blank SMP and PDA-SMP. After the PDA-SMP was modified with PL, a large number of living cells could be observed (Fig. 4d). Moreover, most of the living cells were of irregular shape and some cells were in the process of cell division. Secondly, the PDA-SMP was modified with BSA to improve the anti-platelet adhesion property, which is critical in evaluating the blood compatibility when the material is used for implanted devices. The existence of surface-immobilized BSA was confirmed from the XPS data (ESI Fig. S13†). Herein, we dipped rat platelet-rich plasma (PRP) onto the surface of each sample. Fig. 4e–g show the representative SEM images of rat blood platelet adhesion on the surfaces of the blank, PDA-SMP and PDA-BSA-SMP samples. On the surface of the blank SMP samples, massive adhered platelets could be observed and some of the platelets were severely aggregated. For the PDA-SMP sample, the adhesion was even worse; the adhered platelets could still be observed everywhere and some of them appeared in bigger clusters. A possible explanation for this phenomenon is that the rough morphology and high chemical activity of the PDA coating may lead to a non-specific adsorption or chemical reaction with the rat plasma protein.^43^ In contrast, the platelet adhesion was largely suppressed after the PDA-SMP was further treated with BSA. The BSA layer may act as a barrier preventing PDA from contacting with platelets.^44^ As presented in Fig. 4g, only very few platelet aggregates could be observed on the surface of the BSA treated PDA-SMP sample, which shows a notable improvement in the anti-platelet adhesion properties. These experiments clearly demonstrate that secondary surface modifications are very effective. Meanwhile, the photo-response of the PDA-SMP after secondary surface modification is maintained (ESI Fig. S14 and 15†).

## Conclusions

In summary, different from the previous strategy to make photo-responsive shape memory polymers by incorporating photo-responsive groups or fillers into the material during synthesis, this mussel-inspired approach enables a simple, convenient and green process to endow the normal thermo-responsive SMPs with photo-active properties without influencing the original thermal or mechanical properties at all. The PDA coating shows efficient photo-thermal performance. In addition, using this approach, we can also pattern the surface so as to realize a light-controlled shape programming process, which will largely increase the versatility of SMP applications in practice. Moreover, this PDA coating can also act as a platform for regulating the surface function of SMPs. The secondary modifications of PDA-SMPs with PL and BSA demonstrate a notable improvement in the cell viability and anti-platelet adhesion abilities, respectively, which strongly prove its feasibility. Here, as an example, this approach successfully brings together the four important properties of shape-memory, photo-actuation, shape engineering and surface functionalization in one given SMP material. Since PDA has been recognized to be able to modify almost all materials and be immobilized with various functional groups, this approach can be extended to lots of other SMPs, which offers great opportunities as well as many possibilities in future applications.

## Experimental

### Fabrication of PDA coated SMP films

0.2 g of dopamine and 0.1 g of 2-amino-2-(hydroxymethyl)-1,3-propanediol (Tris) were dissolved in 100 mL of water. The SMP films (prepared according to ref. 36) were immersed in this solution and stirred for 24 h. The resultant PDA coated SMP films were washed with deionized water 3 times and air dried. For the samples used in later cell viability tests, the SMP samples were elongated by 20% of their length before dip coating.

### Measurements

The optical absorption of the PDA coating was measured using UV-visible absorption spectroscopy on a Perkin-Elmer lambda 750 UV/Vis/NIR spectrometer (Waltham, MA, USA). SEM images of the PDA coating were obtained by scanning an FEI Quanta 200 microscope (FEI, Hillsboro, TX, USA). DSC measurements were performed on a TA 2000 analyzer (TA Instruments, New Castle, PA, USA) at a temperature scanning rate of 10 °C min^−1^ under a dry nitrogen atmosphere. Thermogravimetric analysis (TGA) was performed on a TA-Q50 (TA Instruments, New Castle, PA, USA), under an air atmosphere with a heating rate of 20 °C min^−1^ from 25 °C to 800 °C. The tensile tests were measured using dynamic mechanical analysis (DMA) (TA-Q800, New Castle, PA, USA). The storage modulus was measured using DMA (TA-Q800, New Castle, PA, USA) at a multi-strain mode (heating rate 1 °C min^−1^). The reversible photo-response actuation measurement was carried out using DMA (TA-Q800, New Castle, PA, USA) at an iso-strain mode.

### Mechanical modelling

Finite element (FE) modeling was employed to investigate the mechanism of how the PDA pattern guided the bending response. A commercial FE package ANSYS 14.5 was used to create the mechanical model. A rectangular geometry was created with 4-node, two-layer shell elements (Shell 181) to simulate the pre-stretched SMP sheet. The contraction of the shape recovery was simulated by setting the orthotropic thermal expansion to a negative value. High temperature loads were applied to the pattern area to simulate the heat harvested from light. The sheet model was set with two sections, which were defined with different thermal strain values to simulate the actuation difference between the layers due to heat diffusion. As a naive model, the patterned and non-patterned areas were both defined with a linear isotropic Young's modulus (200 Mpa) and Poisson ratio (0.499). More details are shown in the ESI.†

### Secondary modification by polylysine and cell viability test

2 g of polylysine was dissolved in 100 mL of deionized water and the pH was adjusted to 9–10 with 1 M NaOH aqueous solution. Then the PDA-coated SMP film was immersed in the solution and mildly stirred at 35 °C overnight. The resultant PDA-PL-SMP film was washed with deionized water 3 times and air dried. Before the cell viability test, all the samples were sterilized by autoclaving at 120 °C for 30 min.

A549 cells were cultured in DMEM medium supplemented with 10% fetal bovine serum (FBS), 2 mM glutamine, 100 U mL^−1^ penicillin and 100 μg mL^−1^ of streptomycin. The cell culture was maintained at 37 °C in a humidified condition of 95% air and 5% CO_2_ in the culture medium. To perform cell transfection, A549 cells were first seeded on 60 mm dishes. After 24 h of incubation to reach 80% confluence, the culture medium was replaced with a serum-free medium with 20 μL of Lipofectamine 2000 (purchased from Invitrogen) and 8 μg of pEGFP-N1 DNA vector (purchased from Beijing Xin Xing Tang Biotechnology Co., Ltd). The cells were further cultured with Lipofectamine 2000 and the DNA vector for 6 hours before replacing the culture medium with 10% FBS. After 24 hours of incubation, the GFP-transfected cells were harvested and suspended in DMEM medium with a density of 1 × 10^5^ mL^−1^. Then blank SMP, PDA-SMP, PDA-BSA-SMP samples were individually placed in the medium and cultured for 24 hours. After the exposure, all the samples were taken out of the medium, washed with PBS 3 times and then fixed with 4% paraformaldehyde for 10 min at room temperature. Cell images were taken with a laser scanning confocal microscope (LCSM) Zesis 710 3-channels (Zesis, Germany) with an excitation wavelength of 458 nm.

### Secondary modification by bovine serum albumin and platelet adhesion test

0.2 g of bovine serum albumin (BSA) was dissolved into 100 mL of PBS buffer solution (pH = 7.4). Then the PDA-coated SMP film was immersed in the solution and mildly stirred at 35 °C overnight. The resultant PDA-BSA-SMP film was washed with deionized water carefully and naturally dried in air.

The platelet adhesion test was performed according to the paper of Zhu *et al.*^44^ To prepare the platelet-rich plasma (PRP), 10 mL of fresh rat blood was centrifuged at 1500 rpm for 20 min. The rat PRP was obtained from the supernatant. Blank SMP, PDA-SMP and PDA-BSA-SMP samples (1.0 cm × 1.0 cm × 0.015 cm) were placed in a 24-well cell culture plate and 100 μL of PRP was dropped onto each sample. Then the samples were incubated with PRP for 30 min at room temperature (25 °C) and subsequently rinsed with PBS (pH 7.4). The adhered platelets were fixed with 1.0 wt% glutaraldehyde solution for 30 min. The samples were rinsed with deionized water several times and dehydrated in sequence with 30%, 40%, 50%, 60%, 70%, 80%, 90% and 100% (v/v) ethanol/water solution for 30 min each. The resultant samples were naturally dried in air. The surfaces of each sample were observed using scanning electron microscopy (SEM) (FEI Quanta 200, Hillsboro, TX, USA).

The experiments involving the rat blood were performed in compliance with relevant laws and guidelines, which were approved by the regional ethics committee for animal experiments.

## Supplementary Material

SC-007-C6SC00584E-s001
