# Supplementary material for: Polydopamine coated shape memory polymer: enabling light triggered shape recovery, light controlled shape reprogramming and surface functionalization
Source: Chem Sci. 2016 Apr 12;7(7):4741–7. doi: 10.1039/c6sc00584e (PMC6014076; doi:10.1039/c6sc00584e)
Supplement: SC-007-C6SC00584E-s001 [file SC-007-C6SC00584E-s001.pdf]

Electronic Supplementary Material (ESI) for Chemical Science.
This journal is © The Royal Society of Chemistry 2016

Polydopamine coated shape memory polymer: enabling light triggered shape recovery, light controlled shape reprogramming and surface functionalization

Zhen Li,[a] Xiaoyong Zhang,[a] Shiqi Wang,[a] Yang Yang,[a] Benye Qin,[a] Ke Wang,[a] Tao Xie,[b] Yen Wei[a] and Yan Ji*[a]

Characterization of PDA coating

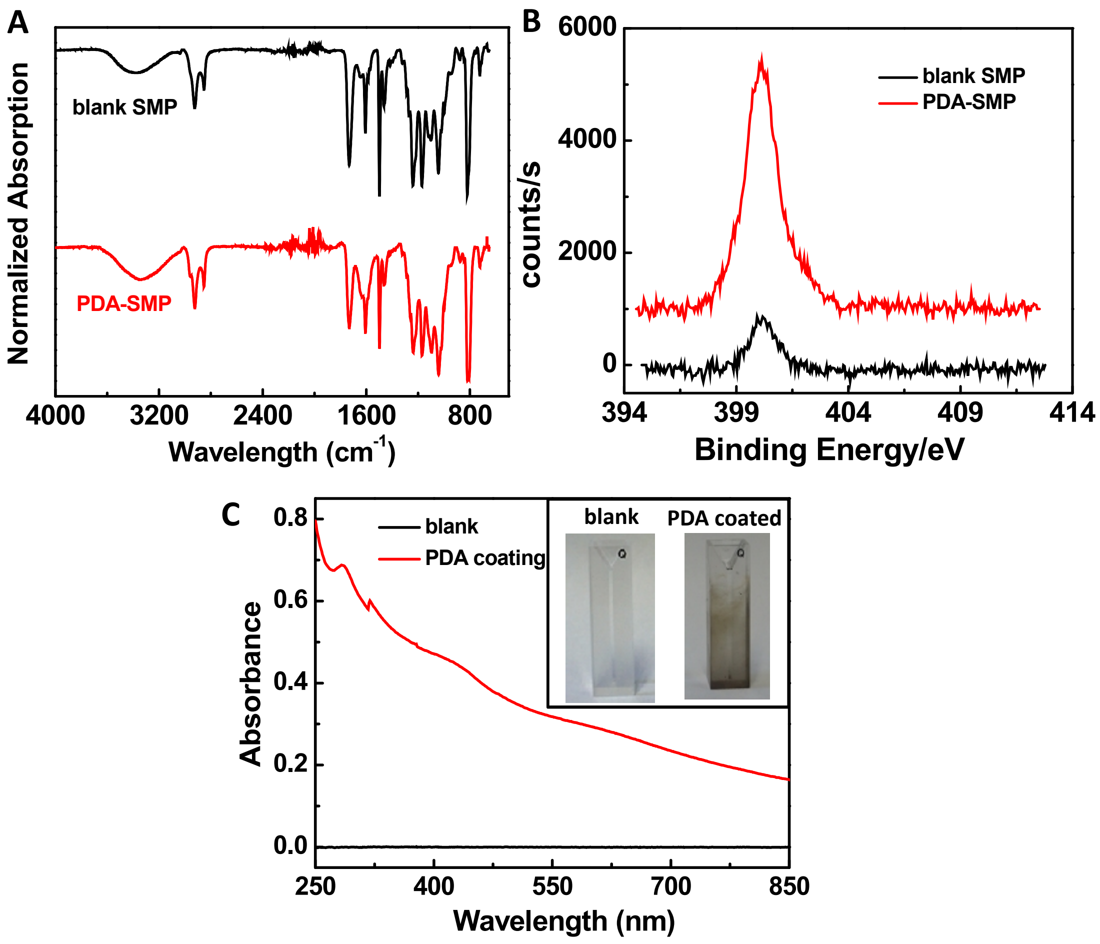

Fig. S1. A) Normalized IR spectra of blank SMP and PDA-SMP. Compared with the blank sample, the IR absorption at 1600 cm^{-1} of the PDA-SMP sample becomes broad due to the increase of C=O group; B) Typical high resolution XPS N 1s spectra of the blank SMP and

PDA-SMP film. Due to the presence of PDA layer, the N element XPS data of PDA-SMP shows a significant increase of N element. C) Optical absorbance spectrum of PDA coating. Inset: optical picture of the blank and PDA coated cuvette. Before measurement, the cuvette was immersed in dopamine solution to obtain a PDA coating on it.

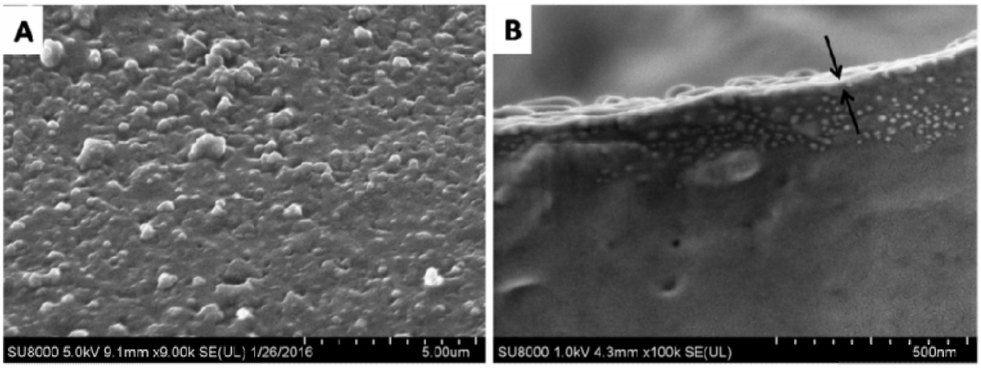

Fig. S2. Scanning microscope images of PDA-SMP sample. A) 45° side view. B) cross section view. The thickness of PDA coating is measured as about 30 nm.

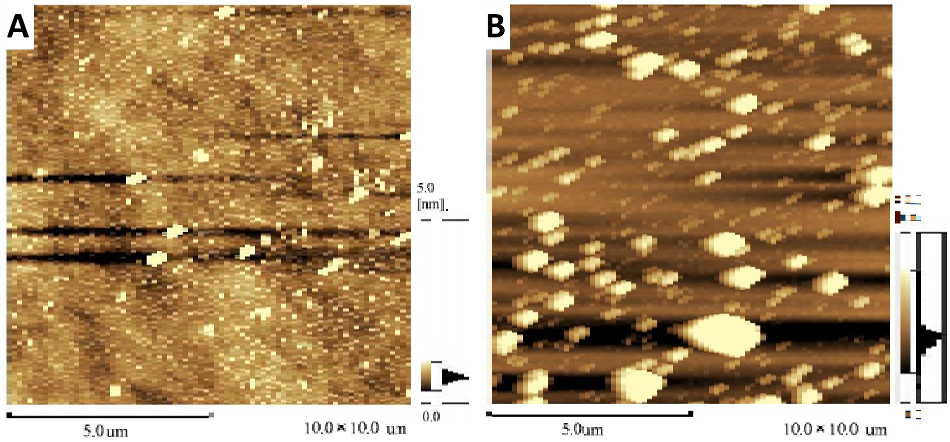

Fig. S3. Atomic force microscope (AFM) images of A) blank silica slice. B) PDA coated silica slice. The thickness of PDA coating is measured as about 35 nm.

Characterization of PDA-SMP

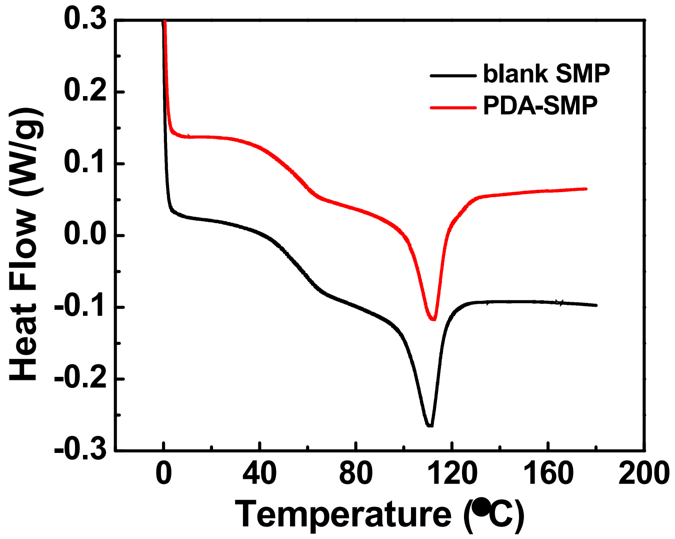

Fig. S4.   DSC curves of the blank SMP and PDA-SMP (10 °C/min).

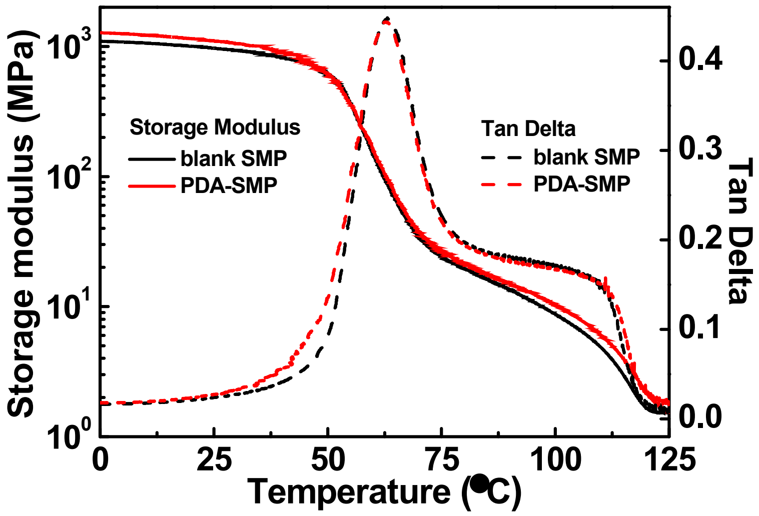

Figure S5. Tensile storage modulus and tanδ trace of blank and PDA-SMP samples measured by DMA (heating rate 1°C/min).

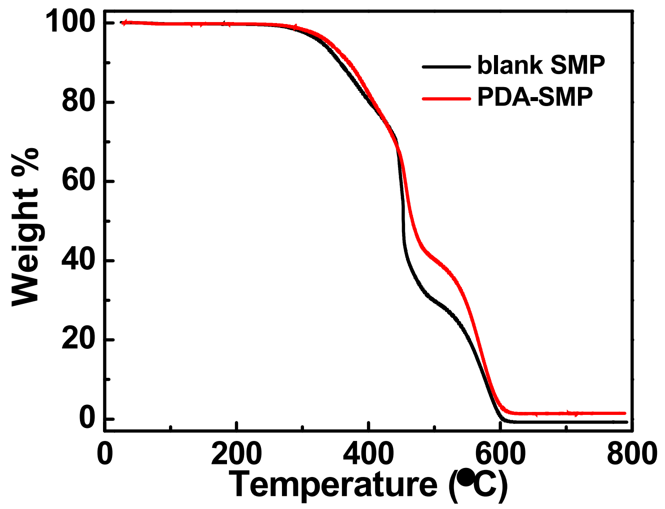

Fig. S6. TGA curves of blank SMP and PDA-SMP samples (heating rate 20 °C/min).

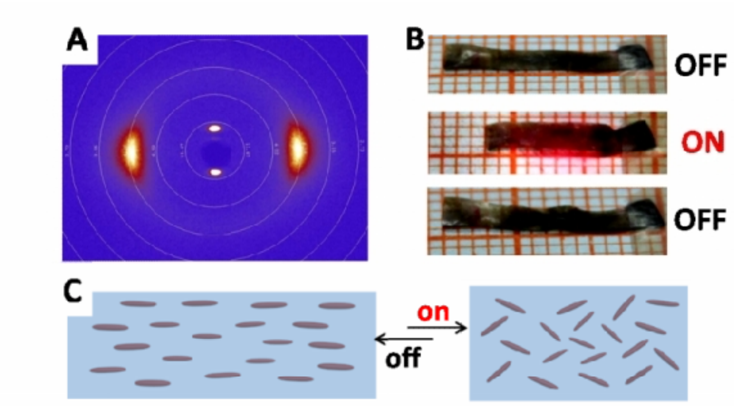

Fig. S7. A) Two-dimensional X-ray image of the aligned SMP sample. B) Optical photograph photo-induced length change of the aligned sample. C) Schematic picture of the order of liquid crystal units with and without irradiation.

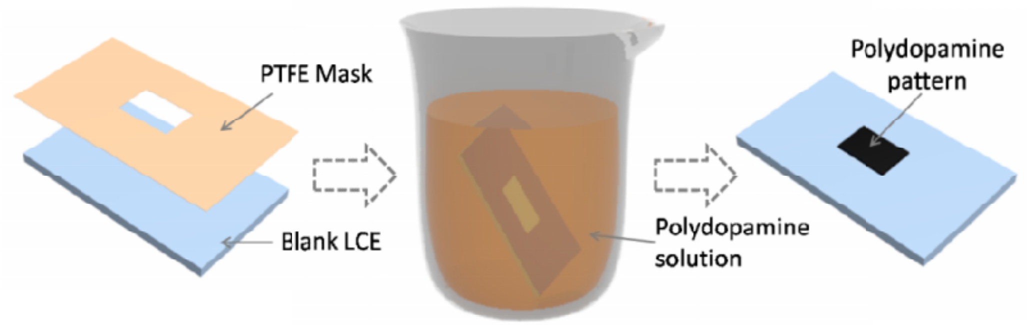

Fig. S8. Manufacturing process of the PDA patterned SMP.

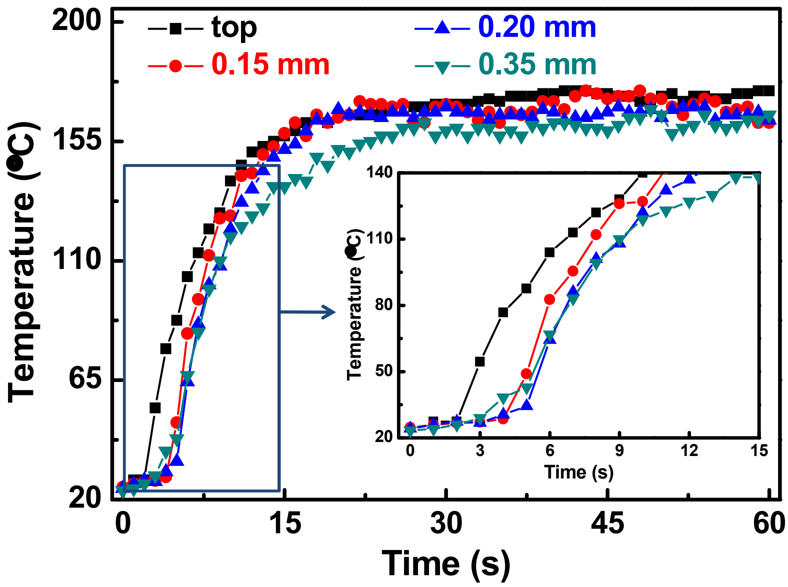

Fig. S9. Local temperature increment of the top and the bottom (different thickness of 0.15 mm, 0.20 mm and 0.35 mm) of a PDA-SMP film under IR irradiation. Inset: temperature increment before 140 °C.

Mechanical Modelling

The element type of the model is 4-node, two-layer shell elements (Shell 181). Rectangular geometries were created to simulate the pre-stretched PDA-SMP sheet. The sections were set as two layers of 0.1 mm thickness. Two material models were created corresponding to the top and bottom layer respectively. Both material models were defined using a liner isotropic Young's modulus (200 Mpa) and Poisson ratio (0.499). The top layer thermal expansion value was set as -0.5 at 80 °C (reference temperature: 40 °C), and that of the bottom layer was set as -0.4 at 80 °C (reference temperature: 40 °C). High temperature (80 °C) loads were applied to the patterned area to simulate the heat harvested from light while lower temperature (40 °C) loads were applied to the blank area.

The boundary conditions for the model conduct were shown in Fig. S10. For the case in Fig. 2a, we tested two different boundaries, thus displacement on one keypoint with all degree of freedom (DOF) and displacement on two sides (four keypoints, one with all DOF and others with only Uz) as shown in Fig. S10A and S10B. It shows that model with the boundary condition on both sides fits the practical sample better while their stress contours of the two boundary conditions exhibit no big difference.

The boundary condition of the case in Fig. 2b was set as a displacement on the middle of the two sides, as shown in Fig. S10E. The boundary condition of the case in Fig. 2c was set as displacements on each line of the rectangle, as shown in Fig. S10G.

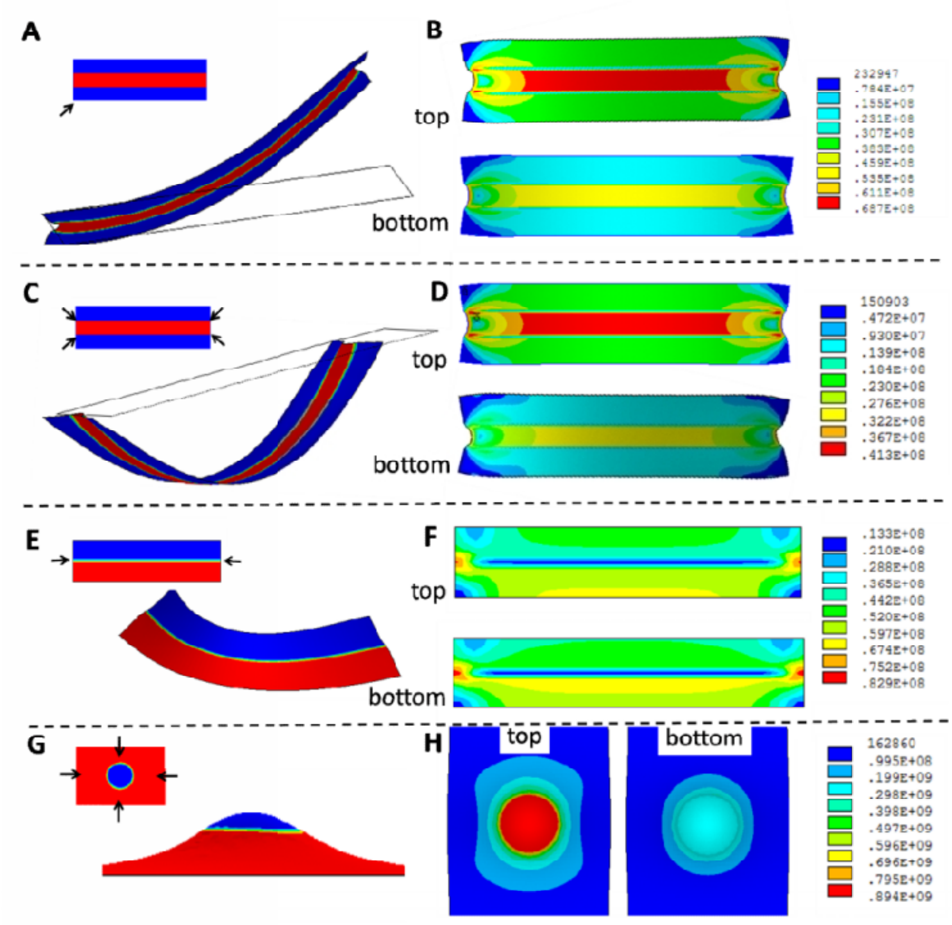

Fig. S10 Sample patterned with a middle line at boundary conditions of displacement A-B) on one keypoint. C-D) on both two sides. E-F) Structure analysis and stress intensity contour of the sample with partially coating. G-F) Structure analysis and stress intensity contour of the coated sample with uncoated circle centre. The stress contour presents that the stress of the top and bottom layer are different, which are assumed as an explanation of the certain deformation.

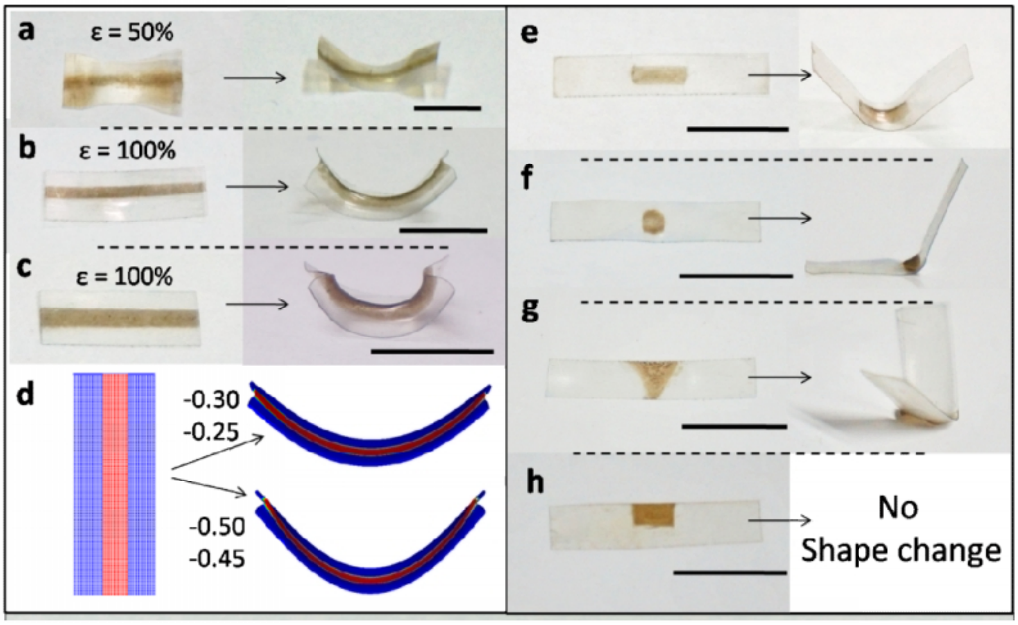

Fig. S11. Shape deformation guided by different PDA patterns. a) A thin-line PDA pattern on a sample with 50% elongation. b) A thin-line PDA pattern on a sample with 100% elongation. c) A wide-line PDA pattern on a sample with 100% elongation. d) Fe model with different loads. e) A rectangle PDA pattern in the middle of a sample with 100% elongation. f) A small circle dot PDA pattern on a sample with 100% elongation. g) A triangle PDA pattern on a sample with 100% elongation. h) A rectangle PDA pattern along side of a sample with 100% elongation. i) Rectangles alternating coated on the top and bottom of the SMP sheet. Scale bar: 1 cm.

As further investigation, we study the cases that influence the bending behavior. As compared in Fig S11a and S11b, the SMP sample with larger elongation will result in bending in larger degrees. Fig S11b and S11c demonstrate the influence from the size of the pattern. With the sample elongation, the wider line will guide a larger bend. By defining the thermal strain of the top and bottom layer as (-0.3, -0.2) and (-0.5, -0.4), the bend in Fig. s11a and S11b were simulated respectively. The simulated results are quite accord with the actual results, confirming proposed mechanism.

We also studied how the pattern shapes influence the deformation. As presented in Fig. S11e and S11f, the rectangle pattern will guide a right angle bend while the small circle dot guides a 120° one, which also explains the deformation in Fig 2g and 2k. As shown in Fig

S11g, the triangle pattern guides a bend both in axial and normal, which explains the helix in Fig 2l. Unexpectedly, we found the rectangle along one side (Fig. S11h) of the sample cannot cause a deformation, showing that the inner stress of the blank regions will also restrict the shape change. This phenomenon explains why the patterned regions didn't recover to the original shape totally, as presented in Fig. 2b.

The purpose of the use of FEM in this manuscript is to support our hypothesis that the certain shape deformation is caused by the thermal gradient across the thickness. However, we found this model could be only used to simulate simple shape deformations and failed to apply it on complex shapes. The possible reasons for this limitation were listed as below:

1. The assumption of a liner material with a Poisson ratio of 0.499 is not in accord with the situation in reality.

2. Shape deformation is dynamic and the constant parameters we used are static.

3. This two-layer shell model may not be sufficient to describe all the factors in reality, such as gravity, density and so on.

Characterization of the secondary modified PDA-SMP

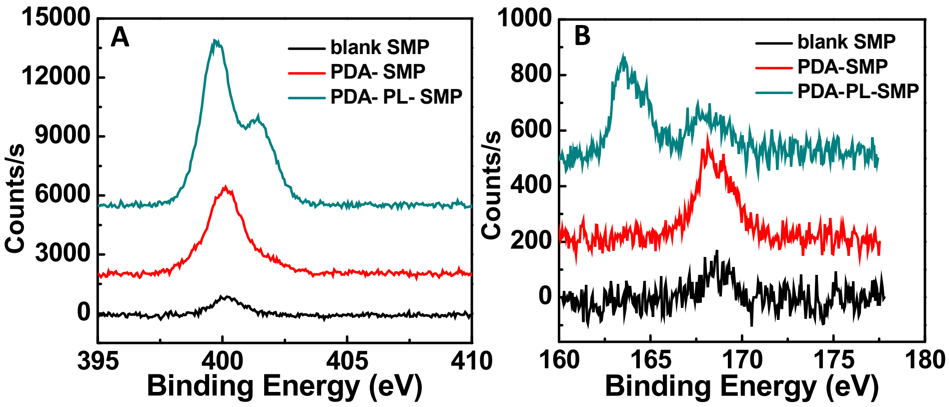

Fig. S12. Typical high resolution XPS spectra of the blank SMP, PDA-SMP and PDA-PL-SMP: A) N 1s and B) S 1s. After secondary modification with PL, a new N spectra peak appears at 402 eV and a new S spectra peak appears at 164 eV, indicating the existence of PL.

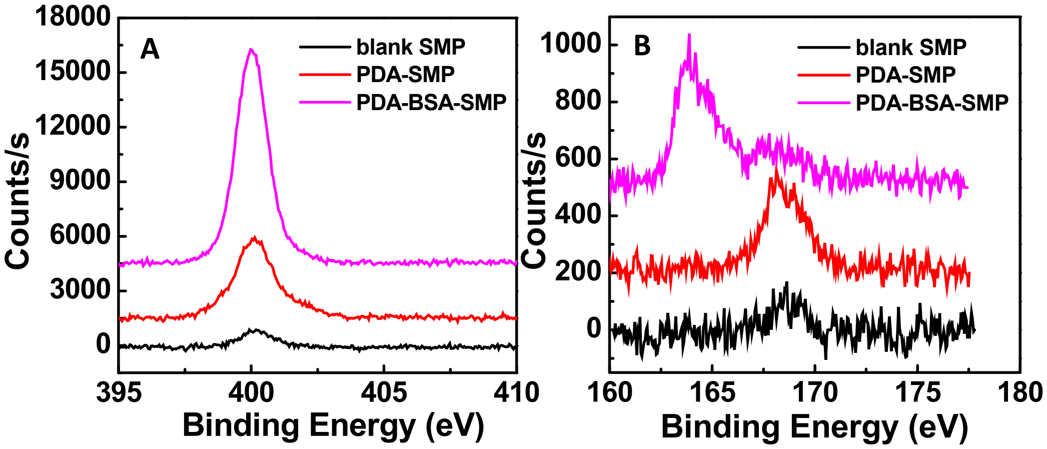

Fig. S13. Typical high resolution XPS spectra of the blank SMP, PDA-SMP and PDA-BSA-SMP: A) N 1s and B) S 1s. After secondary modification with PL, the N spectra peak at 401 eV increases and a new S spectra peak appear at 164 eV, indicating the existence of PL.

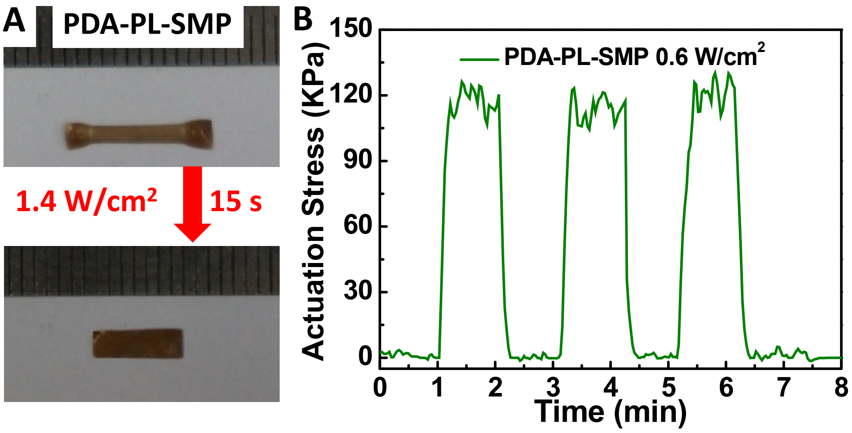

Fig. S14. A) One-way photo-induced shape memory demonstration of PDA-PL-SMP. B) The actuation stress of an aligned PDA-PL-SMP irradiated with 808 nm light at an intensity of 0.6 W/cm^2.

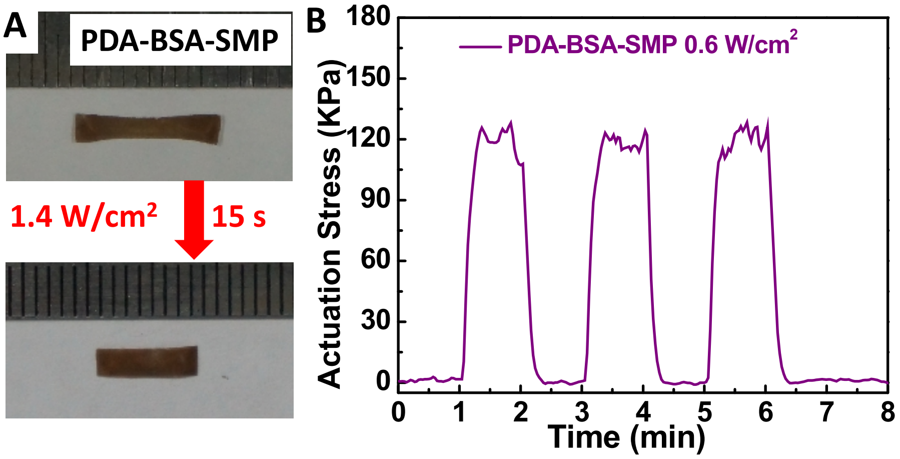

Fig. S15. A) One-way photo-induced shape memory demonstration of PDA-BSA-SMP. B) The actuation stress of an aligned PDA-BSA-SMP irradiated with 808 nm light at an intensity of 0.6 W/cm^2.
